# Supplementary material for: High-Yield and Quantitative Purification Method for HIV Which Minimizes Forces Applied to Virions Utilized to Investigate Maturation of HIV-1 via Cryo-Electron Tomography
Source: Viruses. 2025 Mar 3;17(3):364. doi: 10.3390/v17030364 (PMC11945327; doi:10.3390/v17030364)
Supplement: Supplementary file 1 [file viruses-17-00364-s001.zip › viruses-3467571-supplementary.pdf]

High-yield and quantitative purification method for HIV which minimizes forces applied to virions utilized to investigate maturation of HIV-1 via cryo-electron tomography

Benjamin Preece [1,2,†], Wiley Peppel [1,2,†], Rodrigo Gallegos [1,2], Gillian Ysassi [1,2], Gabriel Clinger [1,2], Nicole Bohn [1,2], Broti Adhikary [3], Luiza Mendonça [3], David Belnap [4,5], Michael Vershinin [6] and Saveez Saffarian [1,2,4,7,*]

[1] Department of Physics and Astronomy, University of Utah, Salt Lake City, UT 84112, USA;
[2] Center for Cell and Genome Science, University of Utah, Salt Lake City, UT 84112, USA
[3] Department of Biochemistry, Molecular Biology and Biophysics, University of Minnesota, Minneapolis, MN 55455, USA;
[4] School of Biological Sciences, University of Utah, Salt Lake City, UT 84112, USA;
[5] Department of Biochemistry, University of Utah, Salt Lake City, UT 84112, USA
[6] Department of Chemistry and Physical Sciences, Nicholls State University, Thibodaux, LA 70301, USA;
[7] Department of Molecular and Cell Biology, University of California, Berkeley, CA 94720, USA
* Correspondence: saveez.saffarian@berkeley.edu
† Authors contributed equally.

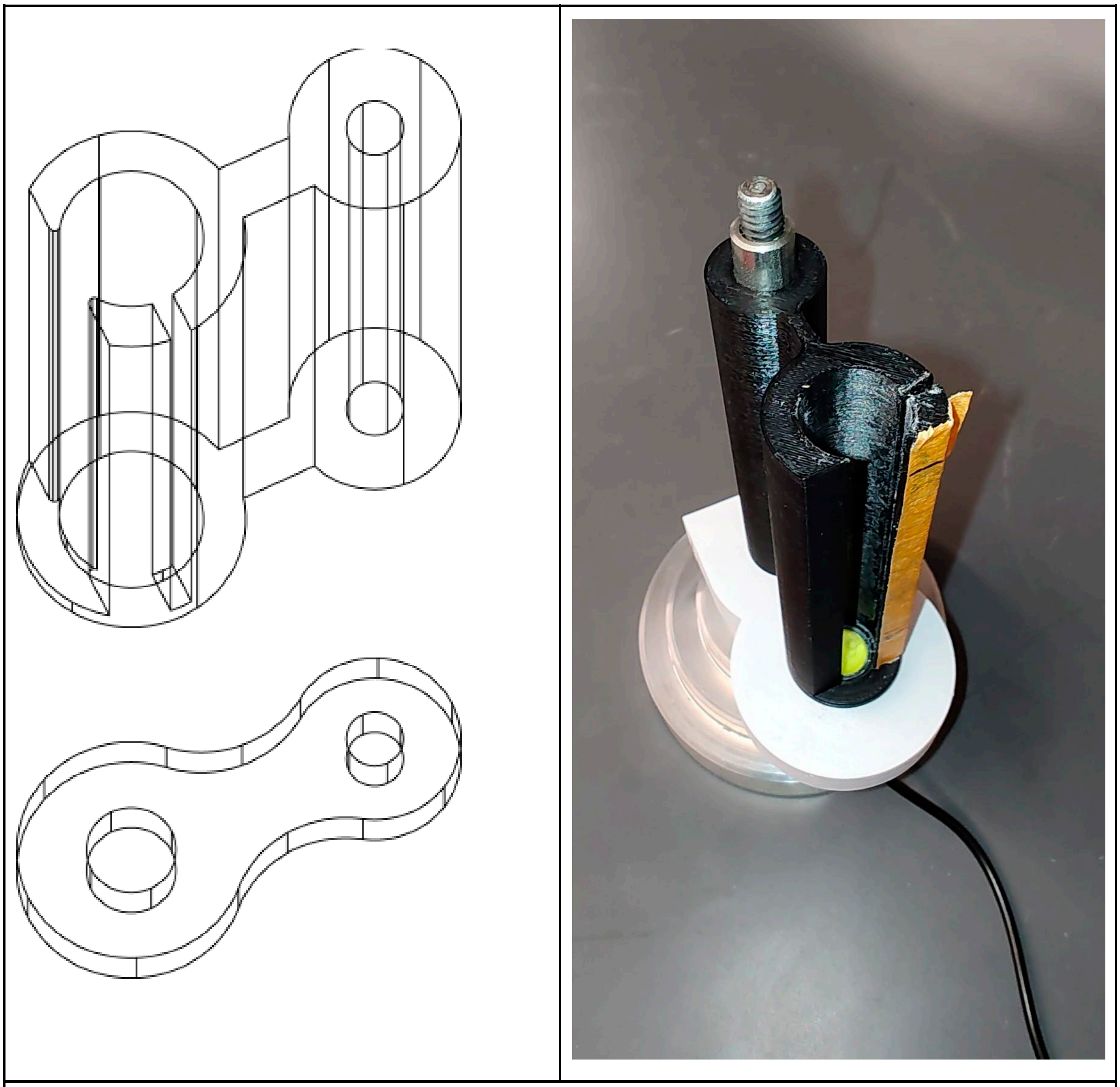

Figure S1: Device constructed for extraction of viral bands from the step gradients. The design allows a 13 ml clear plastic centrifuge tube to be illuminated from the bottom with an LED (The green bulb at the bottom of the chamber) as shown in the image on the right. Left shows the design of the holder from the CAD file used to construct the device. The LED used is manufactured by AUTOMONARCH, brand name CARLITS, lens clear 35 watts.

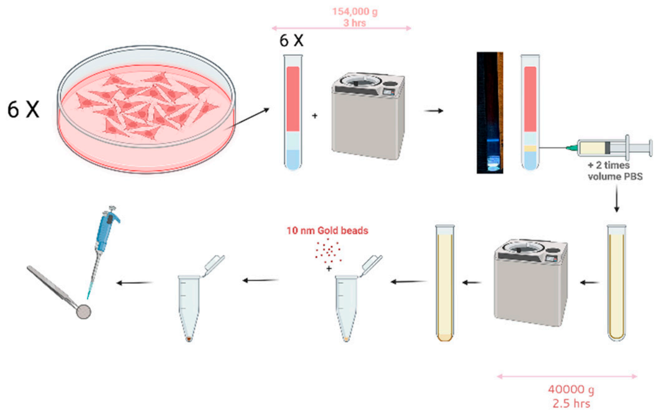

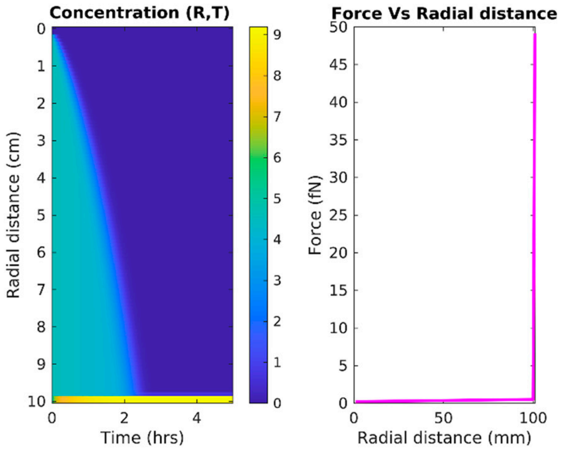

Figure S2:

For Purification Schematic:

Schematic illustration of HIV VLP purification method from six plates of HEK293 cells. Images showing real samples are provided after the step-gradient centrifugation step, the pellet centrifugation step, and after adding the gold nano-particles.

Centrifugation Simulation:

Simulated concentration vs time and force vs radial distance data for VLPs pelleted at 40,000 g in SW41 rotor.

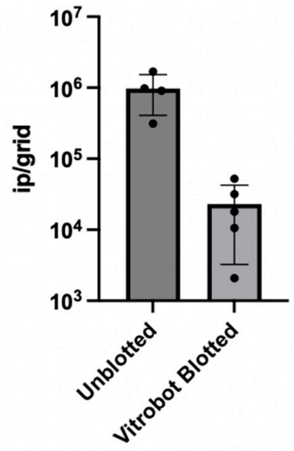

	Unblotted	Vitrobot Blotted
Mean	972656	22925
Std. Deviation	563133	19678
Std. Error of Mean	281566	8800

Figure S3: Measure of virions left in the grid after blotting. Number of infectious particles were quantified using blue-foci assay. 97.7% of viruses are removed by the blotting conditions described in the Methods section.

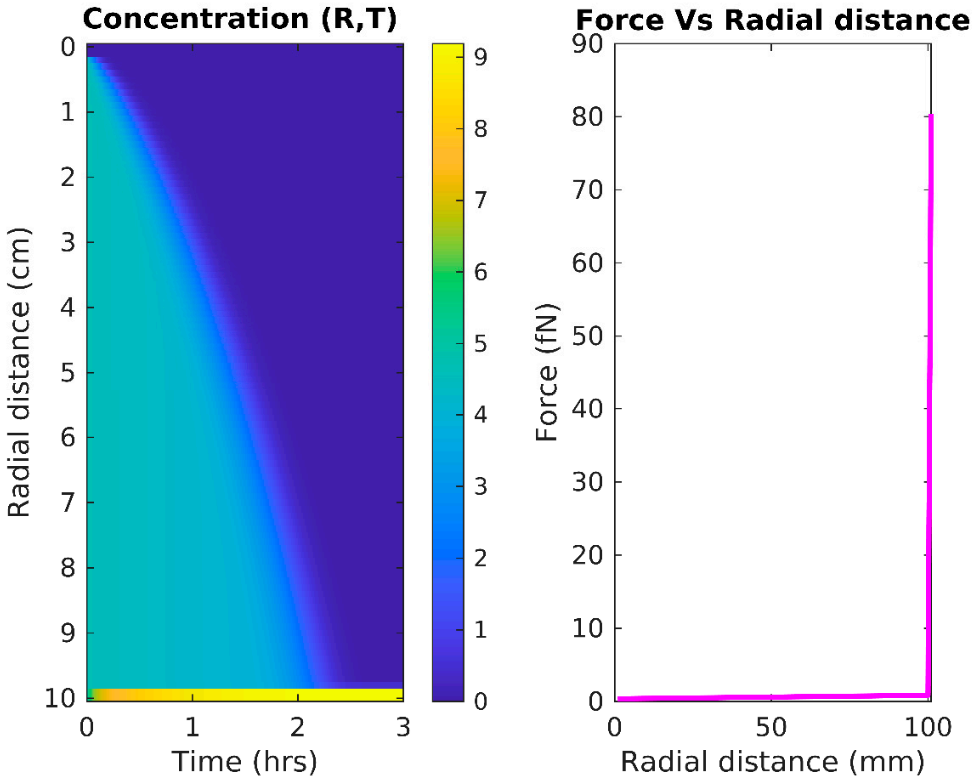

Figure S4: After separation of cells from supernatant, virus-particle-containing supernatants are concentrated by centrifugation through a 20% sucrose cushion at 100,000 × g for 2 h. Our simulation shows this centrifugation using an SW41 Ti rotor.
